# OUTCOMES OF FIT & STRONG! PLUS: A GROUP EXERCISE AND WEIGHT LOSS PROGRAM TO TREAT OSTEOARTHRITIS

**DOI:** 10.1093/geroni/igz038.2274

**Published:** 2019-11-08

**Authors:** Andrew DeMott, Susan Hughes

**Affiliations:** University of Illinois at Chicago, Chicago, Illinois, United States

## Abstract

Overweight older adults with osteoarthritis (OA) face increased risk for disability; however, no evidence-based programs target weight and OA simultaneously. Fit & Strong! (F&S!) is an 8-week evidence-based exercise program for persons with OA that improves lower extremity (LE) strength and mobility out to 18 months. F&S! Plus, a weight loss version of F&S! was tested against standard F&S! in a comparative effectiveness trial. Two and six-month trial outcomes were previously presented, this session will present maintenance outcomes at 12 and 18 months. This trial randomized 413 participants, 210 to F&S! and 203 to F&S! Plus. The mean sample age was 67.9, 86% female, and 92% African American. At 12 months, significant and marginally significant between-group differences favoring F&S! Plus were seen in several outcomes, including weight (p=.049), BMI (p=.04), waist circumference (p=.004), LE physical function (p=.09), 6-minute distance walk (mobility) (p=.08), 30-second chair stands (LE strength) (p=.46), and anxiety & depression (p=.08). At 18 months, only LE strength remained significantly improved for the F&S! Plus group (p=.045), however several within-group improvements remained statistically significant for both groups out to 18 months, including weight, BMI, waist circumference, LE pain & physical function, 30-second chair stands (LE strength), anxiety & depression, and self-efficacy for weight-management. F&S! Plus showed significant improvements over standard F&S! for several outcomes at the conclusion of the intervention (2 months) and many were maintained out to 12 months, with LE strength continuing to 18 months. Both groups showed significant within-group improvements out to 18 months.

